# Dr. Alder, on Quarantines, Contagion, and Fever

**Published:** 1807-12

**Authors:** Thomas Alder


### *To the Editors of the Medical and Physical Journal.*

Gentlemen,

The supporters of febrile contagion and quarantines, may argue that we owe our present exemption from mortal epidemics to the strict enforcement of quarantines? but will they say quarantines have been so performed as to keep all febrile contagion from this island, in every instance, (allowing it for the sake of argument to exist,) or *could be* so performed? Will they affirm quarantines have kept it from America, or the West Indies? Do they not know that fevers were much more extensive, mortal and frequent in the earlier ages of the world, when there were no quarantines than they are now, and can they not turn their attention to the existence and application of the *other* causes of fevers, than their *ideal* contagion?

1. If Gentlemen thought much upon the subject, they would most clearly see the strictest enforcement of quarantines, *could not* keep febrile contagion from the island if it existed: for if every ship, and every article which came in it, weresubjected to forty days, (I might say *forty weeks,*) purification, febrile contagion, (according to the account given of it by those writers who have most supported it,) could not be taken away from such ship: but many ships, and in many articles, which, (according to the language of of the contagionists,) have come from places at all times, which could not but have imbrued them with contagion. And a febrile contagion, (supposing as I before said, any to exist) which wants nothing but a *certain state of the air* to make it as dreadful as any imported from abroad, constantly exists in every part, (that is, in every county, every city, and almost in every town) of our own country; nay, I perhaps might say, we can scarcely turn ourselves in any house, without meeting with it. I apprehend, no just thinking and well-informed medical man, will maintain, these are ideas to which the strict reception of, and reasoning upon, the febrile contagion, doctrines will not lead us!

2. America and other countries were as strict in enforceing quarantines, as we have been when they were most grievously afflicted with fevers, which the advocates for contagion have no reason not to call contagious, so long as they are advocates for febrile contagion.

3. The most extensive and dreadful fevers, (such, for instance, as the febrile contagionists would call the *most dreadful contagious fevers,)* happened at a time when mankind had comparatively no commerce, no intercourse, and no communication: and surely the advocates for quarantine will allow, no communication ought to be more effectual in preventing the spreading of fevers, than the most strict enforcement of quarantines?

4. If Gentlemen would but turn their attention to the *other* causes of fever, or rather to the *only* causes of fever, they would soon see fevers, whether epidemic, endemic, or sporadic, might be caused *without* contagion; and then would soon perceive they *could not be* produced by contagion.

In the time of Morton and Sydenham, I find a fever sometimes inclined to be continued, and sometimes remittent a clearly intermittent, and not very unfrequently conjoined with a rheumatic or pneumonic inflammation, or with coma, was common in the kingdom; sometimes as a sporadic, sometimes as an endemic, and sometimes as an epidemic: indeed I have no doubt it was this fever, which was frequently called the plague itself.

I will not at this distance of time, and at this instant, pretend to specify all the causes of this fever; but I must remark, that in its seasons of prevalence and cessation, it greatly resembled the American and other fevers, and that Britain at that time, with respect to the common causes of fever, (independent of contagion,) was much more similar to to America than it is now. Such places, as in America, have been most attacked with fever, have been near the fountains of bad air, a moisture, or both, just as they have been in Britain, in Egypt, in some of our settlements in Africa, in the West Indies, and in Bengal, and other parts of the East Indies, &c.; those places have suffered from fevers, in proportion as those sources of bad air and moisture were productive, and commonly only in those seasons in which they were productive, i. e. in America and Britain, chiefly in autumn, and towards the latter end of the year:—bnt have too sometimes suffered, when the usual sources of bad air or moisture did not appear to have been more than usually active.

We see from examinations like this, then, that there are two distinct sources of bad fevers, viz. the fountains of putrefaction, &c. and moisture conjoined with the annually unhealthy seasons; and sometimes different from these; (this, I think, the advocates for contagion will allow.) Now, before I saw Mr. Noah Webster's valuable work, I believed this other source of fevers was one which produced nearly the same result, (viz. *bad air*,) and that it often was a volcanic eruption, or some other terrestrial expiration; but I am of opinion, the facts adduced by Mr. Webster, render this beyond all doubt; though I confess he does not seem to have made very clear, or always very right inferences from his own facts; and has, perhaps, on that account, given some *slight* ground for that abuse, which I see has been heaped upon him by the celebrated Dr. Haygarth: abuse, which, however, according to my judgment, is as cruel as injurious to literature and to society, and is in fact, in all very essential respects, as *unjust* as any that I ever saw applied to a literary character.

The bad air, which formerly gave rise to the continual aggravation of fever in this country, seems to have been owing, (independent of that caused more immediately by the season,) in a a great measure, to the great quantity of undrained land, which then was in it, to the small unventilated houses formerly used, and to the crowded dirty state of the metropolis: drainage of moist land, in private farms, was little practised; the great fens, in the counties of Cambridge and Lincoln, and to the East of London, were left to nature; and the metropolis, till the great fire, was in a great part of it, a pile of huts huddled together, and very dirty. But the bad air, which occasioned an irregular and more extensive epidemic, seems to have come chiefly from some terrestrial expirations; this, however, was was frequently joined with the bad air above noticed, and some from other sources not yet particularized. That terrestrial expirations have caused much disease in this kingdom, as well as in other, is evident from many circumstances; but especially from the great eruptions which have taken place in mount Hecla, and the diseases which have immediately followed, though sometimes preceded them, from black snow, from such eruptions having reached sooner in summer, from the quantity of fish which have died on the British Coasts, and in the Baltic, and from the remains of Volcanos being still visible in Scotland.

The way in which my latter assertion is proved by these facts, is, perhaps, more just than at first sight obvious: it is this,—the eruptions of mount Hecla having been great and numerous, have sent into the air immense quantities of morbiferous gases;—and that these gases might reach Britain, is shewn by snow made black by them reaching it *in summer*; and also by epidemics breaking out.—When epidemics, or neighbouring endemics, have followed volcanic eruptions, they have by many been attributed to them; but Mr. Webster has very culpably been accused of attributing them not to the eruptions, but to the cause of the eruption, when they have happened *before the eruption*,—as morbiferous gases usually exhale in great abundance, *long before* an eruption takes place. The prodigious quantity of fish, which has died near the time of some terrestrial explosion, has usually been attributed to the concussion and disturbance of the water; but it has clearly been occasioned by the effusion of lethiferous gases into it. The remains of Volcanoes, in Scotland, prove we have had noxious terrestrial expirations, nearer home than mount Hecla is.

If the expressions contained in this, shall appear too concise, I will endeavour to be more full afterwards.

I am, &c.

THOMAS ALDER.

*November* 2, 1807.

Account

